# COVID-19 and Acute Coronary Syndromes: Current Data and Future Implications

**DOI:** 10.3389/fcvm.2020.593496

**Published:** 2021-01-28

**Authors:** Matteo Cameli, Maria Concetta Pastore, Giulia Elena Mandoli, Flavio D'Ascenzi, Marta Focardi, Giulia Biagioni, Paolo Cameli, Giuseppe Patti, Federico Franchi, Sergio Mondillo, Serafina Valente

**Affiliations:** ^1^Department of Medical Biotechnologies, Division of Cardiology, University of Siena, Siena, Italy; ^2^University of Eastern Piedmont, Maggiore della Carità Hospital, Novara, Italy; ^3^Department of Clinical Medical and Neurosciences, Respiratory Disease and Lung Transplantation Section, Le Scotte Hospital, University of Siena, Siena, Italy; ^4^Department of Medical Biotechnologies, Anesthesia and Intensive Care, University of Siena, Siena, Italy

**Keywords:** SARS-CoV2, myocardial injury, NSTEMI, STEMI, acute coronary syndromes, COVID-19

## Abstract

Coronavirus disease-2019 (COVID-19) pandemic is a global healthcare burden, characterized by high mortality and morbidity rates all over the world. During the outbreak period, the topic of acute coronary syndromes (ACS) has raised several clinical issues, due to the risks of COVID-19 induced myocardial injury and to the uncertainties about the management of these cardiologic emergency conditions, which should be organized optimizing the diagnostic and therapeutic resources and ensuring the maximum protection to healthcare personnel and hospital environment. COVID-19 status should be assessed as soon as possible. Moreover, considerably lower rates of hospitalization for ACS have been reported all over the world, due to patients' hesitations to refer to hospital and to missed diagnosis. As a result, short- and long-term complications of myocardial infarction are expected in the near future; therefore, great efforts of healthcare providers will be required to limit the effects of this issue. In the present review we discuss the impact of COVID-19 pandemic on ACS diagnosis and management, with possible incoming consequences, providing an overview of the available evidence and suggesting future changes in social and clinical approach to ACS.

## Introduction

### Background

Coronavirus-2019 (COVID-19) outbreak is currently the most discussed public health issue, caused by the highly infectious severe acute respiratory syndrome coronavirus 2 (SARS-CoV-2). COVID-19 was declared a pandemic by the World Health Organization in early March 2020 and it was characterized by an exponential rise in contagions worldwide, with continuously increasing number of victims (1). The typical clinical spectrum of COVID-19 includes fever, cough, myalgia, dyspnea (2), with frequent progression to pneumonia, which in one third of the cases eventually leads to acute respiratory distress syndrome (ARDS), of which another third warrant critical care (3). Therefore, prevention and treatment of COVID-19 are currently the primary focus of clinical and scientific debates. However, acute coronary syndromes (ACS) management during this emergency period is gaining growing interest, yielding many scientific researches as well as national and international societies consensus documents, stimulated by four major concerns:

- an increase in short-term risk of myocardial injury and infarction has been reported, particularly for patients with underlying CAD and/or pro-inflammatory cardiovascular risk factors (such as diabetes mellitus, hypertension, and obesity);
- differential diagnosis between non-COVID ACS and COVID-19 induced acute myocardial injury (COVID-AMI), and within COVID-AMI, among myocardial infarction (MI), acute viral myocarditis, stress cardiomyopathy, is currently challenging, due also to the restricted availability of diagnostic tools;
- a sensible reduction of the rates of ACS has been recorded all over the world (4), probably not only as a consequence of lower patients' referral to the emergency department (ED), but also of misdiagnosis;
- lack of preparation and standardized protocols to balance between timely management of ACS and protection of healthcare personnel and hospital environment has provoked delays in the treatment of high-risk ACS; this fact, in conjunction with the previous point, has led to an increased incidence of short-term MI complications and estimated higher long-term MI complications, which will probably require changes in public health resources and system.

### Aims

In the present review we sought to address these four important issues, discussing the earliest evidence and recommendations present in literature, and providing hints and previsions for the future, in order to prepare clinicians and solve their uncertainties on the matter of ACS during and after COVID-19 pandemic.

## Acute Myocardial Injury Triggered by COVID-19

The development of myocardial injury is not uncommon among patients with COVID-19 and correlates with disease severity. In fact, a meta-analysis involving 1,527 COVID-19 patients revealed that at least 8% of the patients had acute myocardial injury and that the risk of myocardial injury is 13-fold higher in patients with severe clinical presentation (5).

COVID-AMI has been defined as the elevation of high-sensitivity cardiac troponin (hs-cTn) above the 99th percentile of its upper limit of normal or evidence of new electrocardiographic (ECG) or echocardiographic abnormalities (6). In fact, the presence of increased levels of hs-cTn was found to be an independent predictor of disease severity and mortality rate in COVID-19 (7) even after adjustment for baseline characteristics and medical comorbidities, also showing an association with the need for intensive care unit (ICU) admittance (RR 13.48, 95%CI 3.60 to 50.47, *p* = 0.0001) (5).

## Differential Diagnosis

There are different potential etiologies of COVID-AMI: ACS due to plaque rupture or thrombosis (type I MI) or to supply-demand mismatch (type II MI), myocardial injury due to disseminated intravascular coagulation (DIC), and non-ischemic injury (myocarditis, stress-induced cardiomyopathy, cytokine release syndrome, acute pulmonary embolism). Each one is the result of a direct or indirect effect of severe viral infection, as explained in Table 1. It is essential to recognize ACS and ACS-mimicker in order to provide an adequate treatment and avoid additional risks (e.g., fibrinolysis in case of myocarditis or stress-cardiomyopathy would expose patients to bleeding risk and eventual invasive coronary angiography (ICA) for unresolved ST-elevation rather than being beneficial) (6).

**Table 1:** Different etiologies and hypothesized mechanism of COVID-induced myocardial injury.

**Type of myocardial injury**	**Possible mechanism**	**Clinical consequences**	**Available evidence**
Type 1 myocardial infarction	*Systemic inflammatory response syndrome*: ↑risk of plaque rupture and thrombus formation **Cytokine storm** due to imbalanced TH1/TH2 response ⇒**DIC** [*71.4% non-survivors vs. 0.6% survivors* (8)]: MOF	STEMI or NSTEMI (9) **Thrombosis** of coronary epi- and subepicardial arteries ⇒focal myocardial necrosis and dysfunction (10)	Bangalore et al. (11) Xhuan et al. (12) Tang et al. (8) Sugiura et al. (10)
Type 2 myocardial infarction	**Myocardial oxygen imbalance** (↑demand for sepsis state, not satisfiable for COVID-19 induced hypoxaemia and vasoconstriction)	Severe myocardial ischaemia, ++ in patients with underlying CAD	Li et al. (5) Shi et al. (13) Guo et al. (14)
Venous thromboembolism	Hypercoagulable status + active inflammation + propensity for DIC + prolonged immobilization + oxidative stress + endothelial dysfunction + increased platelet reactivity + mechanical ventilation + liver dysfunction + central venous catheters + nutritional deficit	↑D-dimer *(>1μg/mL on admission ⇒↑ in-hospital death)*, FDP, fibrinogen Pulmonary embolism or deep venous thrombosis *[22.7% non-ICU and 27% in ICU patients* (15)]	Tang et al. (10) Han et al. (15) Klok et al. (16)
Acute myocarditis	*Indirect mechanism:* innate immunity activation ⇒inflammatory cascade and exaggerated cytokine release *Direct mechanism*: ACE2 receptor (used by SARS-CoV2 for binding, overexpressed in diseased hearts)	STEMI-like presentation with myocardial degenerative changes and necrosis	Zhou et al. (17) Yao et al. (18) Beri et al. (19) Tavazzi et al. (20) Hu et al. (21) Zeng et al. (22) Sala et al. (23)
Stress cardiomyopathy	Infective +/- emotional trigger ⇒catecholamine induced myocardial stunning or macro- and micro-vascular spasm	Tako-tsubo syndrome	Moderato et al. (24) Meyer et al. (25) Chadha et al. (26)

*ACE2, angiotensin-converting enzyme-2; CAD, coronary artery disease; DIC, disseminated intravascular coagulation; ICU, intensive care unit; MOF, multi-organ failure; NSTEMI, non ST-elevation myocardial infarction; SARS-CoV-2, severe acute respiratory syndrome coronavirus 2; STEMI, ST-elevation myocardial infarction; TH1, T-helper lymphocytes 1; TH2, T-helper lymphocytes 2; VTE, venous thromboembolism*.

*+, plus; ++, above all; ↑, higher*.

### Differential Diagnosis: First Contact With Patients

The distinction between primary ACS and COVID-AMI for outpatients referring to ED would be crucial for the subsequent patient management, not only for treatment but also for the safety measures to employ (i.e., isolation, use of adequate personal protective equipment [PPE]). In accordance to the European Association of Percutaneous Cardiovascular Interventions (EAPCI) recommendations (27), for patients with suspected ACS, the likelihood of COVID-19 status should be assessed through accurate clinical interview, investigating the presence of typical symptoms (e.g., fever, cough, dyspnea, cold) or contacts with COVID-19 infected, together with the execution of nasal and/or oropharyngeal swab for SARS-CoV2 Nucleic Acid test as soon as the patient arrives in the ED, if possible. Fast-track pathways for the exclusion of COVID status would expedite the management of these patients. Until the result of the swab is ready, each patient should be considered as COVID infected; this is also valid for STEMI patients who are transferred to the catheterization laboratory (Cath-lab) before having the results. Healthcare workers and patients must always wear at least droplets PPE (i.e., surgical mask, gloves, cup, goggles, and single-use gown for clinicians, surgical mask and gloves for patient). Moreover,

- in case of patients with asymptomatic/negative anamnesis and negative SARS-CoV2 Nucleic Acid test the *common ACS-pathway* should be followed;
- in case of patients with symptomatic/positive anamnesis and negative SARS-CoV2 Nucleic Acid test, the *swab should be repeated;*
- in case of positive SARS-CoV2 Nucleic Acid test, patients are considered as COVID infected, healthcare professionals must wear total-protection PPE (i.e., cup, facial protection, waterproof single-use gown and gloves) and filtering face piece class 3 (FFP3) or N95 mask.

Based on our clinical experience, we suggest that it could be reasonable, while awaiting swab results, prioritize timely treatment in high-risk patients, considering them as COVID-19 infected in order to provide timely treatment and perform ICA, whenever indicated, using airborne PPE (coverall or disposable gown, gloves, headcover, eye shield, FFP3/N99 respirators masks, and shoe covers); then, after revascularization, assess COVID-19 status in order to organize hospitalization in a dedicated ward or isolation in coronary care unit, and subsequent healthcare workers' use of different types of PPE.

### Differential Diagnosis in COVID-19 Patient

Differential diagnosis of COVID-AMI really became a challenge for clinicians. Commonly, a rise and/or fall of hs-cTn is not sufficient to ensure the diagnosis of myocardial infarction, but it should also be corroborated with clinical judgment, symptom and signs, ECG changes, and imaging studies (28). As recent documents of the European Association of Cardiovascular Imaging (EACVI) and the American College of Cardiology (ACC) highlighted, this is especially valid in case of COVID-19, considering that cardiac enzymes elevation could either be secondary to non-specific raise during COVID infection or to other acute pathologic complications (e.g., sepsis, acute kidney injury, stroke) (29, 30). Moreover, as troponin elevation in patients with COVID-19 infection seems to be lower than in most cases of ACS or acute myocarditis, EAPCI suggests considering marked elevation (e.g., >5 times the upper normal limit) in a patient who is not critically ill to suspect COVID-AMI (27).

As a matter of fact, the access to diagnostic resources is currently limited since, considering the high infective power of SARS-CoV2, performing unnecessary imaging tests should be avoided in order to limit healthcare personnel and devices exposure to the risk of contamination (31).

Sometimes, COVID-19 presentation could entail cardiovascular symptoms rather than fever, cough, dyspnoea, as shown in a small Italian report with 81% of patients presenting ST-elevation MI (STEMI) as COVID-19 first manifestation, of whom 78.6% referring to ED with acute chest pain. Interestingly, only 39.3% demonstrated absence of obstructive coronary artery disease (32). In fact, the EACVI recommendations on the use of cardiac imaging during COVID-19 pandemic suggest considering the optimization of computed tomography (CT), often used to confirm of COVID-pneumonia, with the addition of coronary CT methods to exclude ACS in case of raised troponin (30). Similarly, the use of CT completed with contrast enhanced sequences has been proposed by Hendren et al. to exclude acute myocarditis avoiding the additional use of cardiac magnetic resonance (CMR) and invasive endomyocardial biopsy, since patterns of delayed myocardial enhancement consistent with acute myocarditis revealed by cardiac CT have also been described (33).

As regards patients hospitalized for COVID-19 with suspected ACS, EACVI recommends to evaluate the pre-test probability (PTP) based on symptoms, ECG signs, age, sex, previous history, and cardiovascular risk factors, to use coronary CT angiography for intermediate PTP, and to reserve ICA only for cases with very high PTP or STEMI, high-risk non-STEMI (NSTEMI) or crescendo angina (34).

**Figure 1:**
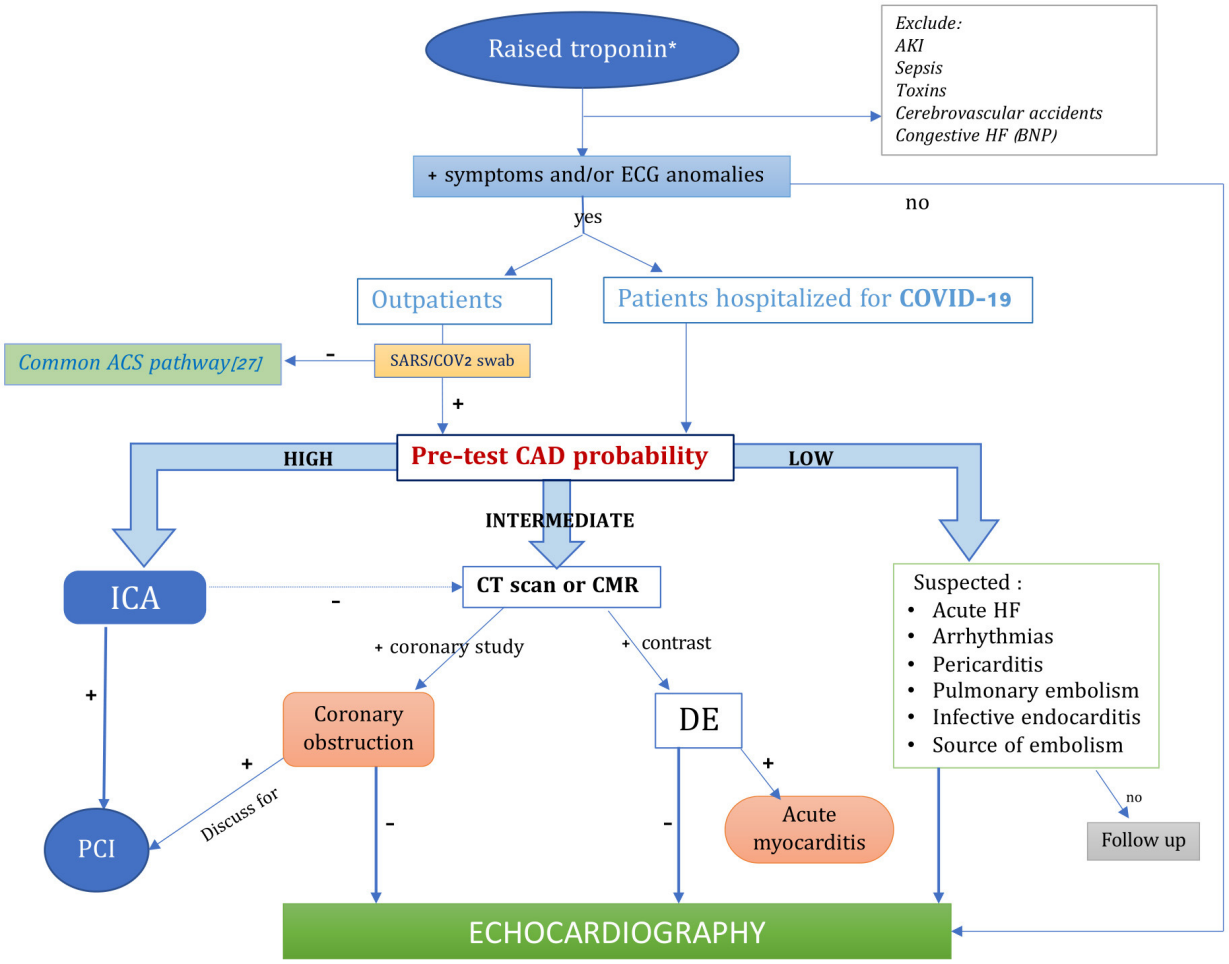
Algorithm for the diagnosis of COVID-induced acute myocardial injury optimizing the available imaging techniques. *hs-cTn>99th percentile of its upper normal limit, or >5 times the upper normal limit in COVID patients. ACS, acute coronary syndromes; AKI, acute kidney injury; BNP, brain natriuretic peptide; CMR, cardiac magnetic resonance; CT, cardiac tomography; DE, delayed enhancement; HF, heart failure; ICA, invasive coronary angiography; PCI, percutaneous coronary intervention.

A schematic representation of the suggested pathway for differential diagnosis of COVID-AMI preventing from wasting unnecessary diagnostic resources is available in Figure 1. In that regard, two important messages deriving from the international societies' recommendations (27, 29, 30, 34), both for outpatients referring to ED and for hospitalized patients, should be highlighted:

- **ICA** should be performed only in patients with suspected type 1 MI (27) and who are expected to derive meaningful changes in outcome from invasive management; therefore, patients with high level of comorbidities, poor quality of life, and frailty should be early assigned to medical therapy, since additional investigations are futile;
- the use of **echocardiography**, which has always been regarded as a “gatekeeper” for differential diagnosis of cardiovascular disease, should be reconsidered in this emergency period. Transthoracic echocardiography should not be routinely performed if patients are asymptomatic and stable, but it remains the first line approach in patients with high suspicion of COVID-AMI, in order to address diagnosis (35). Given its high aerosol-generating procedure, the use of transoesophageal echocardiography should be restricted to the selected cases of poorly feasible or informative transthoracic echocardiography, and when it would lead to change and optimization of the patient's management; when necessary, this procedure must be performed with FFP3 or N95 equipment.

Bearing all these recommendations and the possible poor availability of advanced imaging methods in some center, also due to the overwhelming requests of CT scan, for the purpose to determine the presence of an atypical COVID presentation with ACS, we would like to highlight the importance of performing accurate anamnesis, investigating symptoms occurrence and timing; a thorough ECG observation, seeking for ischemic abnormalities corresponding to coronary regions; rely on the dosage of troponin, after excluding troponin-affecting comorbidities which could act as confounders. In cases of extreme uncertainties, echocardiography should be applied with the use of appropriate PPE (Figure 1).

### In-hospital ACS Management During COVID-19

#### Outpatients

The best therapeutic strategy for patients with ACS during the pandemic has been extensively discussed. Even though in early Chinese algorithms primary PCI was sacrificed in favor of the protection of healthcare personnel from contagion, opting for rapid testing for COVID-19 infection and immediate fibrinolysis, European societies recommend a halfway approach (34, 36). Accordingly, as stated in the EAPCI document on invasive management of ACS during COVID-19 (27), the COVID-19 infective danger should not change the first-line therapeutic approach to STEMI. Primary percutaneous coronary intervention (PCI) remains the standard of care for STEMI patients referred to Hub centers or transferred rapidly from non-PCI centers within 120 min from the first medical contact. For patients in whom a rapid reperfusion with primary PCI is not feasible, initial fibrinolysis is recommended, followed by consideration of transfer to a PCI center. More specifically, the consensus statement from the Society for Cardiovascular Angiography and Interventions (SCAI), ACC and the American College of Emergency Physicians (ACEP) suggests that for *STEMI patients with positive SARS-CoV2 swab* referred to a Spoke center, the transfer to a PCI center should be discussed, possibly preferring to perform fibrinolysis within 30 min of STEMI diagnosis, and eventually transfer to Hub Center for rescue PCI if needed (37), where this should be performed by experienced operators equipped with high-level PPE in dedicated rooms.

For NSTEMI management an approach based on individual risk is recommended (27):

- *very high risk NSTEMI* patients should follow a similar management of STEMI;
- *high risk NSTEMI* patients should follow medical treatment while waiting for SARS-CoV2 test results and planning an early invasive therapy, possibly <24 h; in case of positive test, the patients should undergo ICA in a COVID-19 hospital;
- *low risk NSTEMI* could be firstly evaluated non-invasively, in order to exclude alternative etiology to type 1 MI, using coronary CT, if possible; if low risk is confirmed, they should follow conservative strategy.

Table 2 summarizes the criteria for risk stratification of NSTEMI patients based on the newest European Society of Cardiology (ESC) guidelines (38).

**Table 2:** Risk stratification for non-ST-elevation myocardial infarction (NSTEMI) treatment (38).

**Very high risk**	**High risk**	**Low risk**
- Hemodynamic instability - Cardiogenic shock - Recurrent/refractory chest pain despite medical treatment - Life-threatening arrhythmias - Mechanical complications of myocardial infarction - Acute HF related to NSTEMI - ST-segment depression > 1 mm in 6 leads + ST-segment elevation in aVr and/or V1	- NSTEMI diagnosis already established - Symptomatic/asymptomatic - dynamic new (or presumably new) contiguous ST-T segment changes - Resuscitated cardiac arrest without ST-segment elevation or cardiogenic shock - GRACE risk score > 140	No recurrence of symptoms and none of the *very high* or *high-risk* criteria. Also includes patients with: - History of revascularization - Early post-infarction angina - LVEF <40% or congestive HF - GRACE risk score 109–140 - Diabetes mellitus Ruled out based on troponin levels

*HF, heart failure, NSTEMI; LVEF, left ventricular ejection fraction; non-ST-elevation myocardial infarction*.

In case of necessary ICA approach, preventive strategies are of outmost importance to ensure protection to healthcare personnel and their relatives, hospital environment, and also other patients.

As regards high-risk patients whose COVID status is unknown, as soon as the patient arrives in the Cath-lab, vital signs should be assessed (with particular attention to body temperature and arterial oxygen saturation). Furthermore, blood gas analysis and biologic specimens (swab) collection for COVID-19 test should be performed using the necessary PPE according to the severity of respiratory symptoms (39):

- Low COVID-19 risk: surgical mask.
- High COVID-19 risk: PPE with FFP2 or FFP3 mask, depending on the gravity of respiratory impairment of the individual patient.

Operators should follow precise protocols of dressing/undressing (40) and, after the procedure, in patients with unknown or positive SARS-CoV2 Nucleic Acid test a sanitization of the Cath-lab is mandatory.

#### Inpatients

As for patients already hospitalized in a *COVID-Unit* with *suspected STEMI*, the risk and benefits of a possible coronary revascularization should be evaluated, weighting the individual patients' clinical conditions and comorbidities and the risks related to the transport in the Cath-lab. In case of risks overweight, fibrinolysis could be considered as an alternative to PCI (41, 42). However, the increased hemorrhagic and DIC risk in COVID-19 patients, especially those with severe conditions, should be considered.

#### Fibrinolytic Strategy

Even if bigger evidence is required in this field, the use of fibrinolysis as an alternative to PCI seemed to reach comparable results for in-hospital and 30-day clinical outcome (all-cause death, cardiac death, stroke, re-infarction/coronary re-occlusion, and revascularization) in patients during the COVID-19 pandemic with absence of major bleeding (43) and was proposed by several authors as a reasonable alternative to PCI, providing spare of medical resources (e.g., PPE and workflow) and of healthcare professionals exposure to the risk of contagion (41, 44, 45). However, we suggest that (1) the well-known superiority of PCI to definitely restore blood flow and in reducing mortality, re-infarction, or stroke (46); (2) the risk of early re-thrombosis of the culprit lesion requiring rescue PCI if sufficient anticoagulation is not reached after the fibrinolytic treatment, resulting in longer hospitalization and possible complications; (3) the fatal/non-fatal bleeding risk of fibrinolysis itself (particularly if performed in patient with “STEMI-mimicker”) should be taken into account both in COVID-19 and non-COVID-19 patients; therefore, in our view, fibrinolysis-lone strategy should be considered only in case of higher risks connected to patients' transfer to PCI-center or to the Cath lab outweighing incremental benefits of PCI, or in case of impossibility to provide timely PCI. Importantly, the bleeding risk of the single patient should be evaluated in the decision-making between primary PCI and fibrinolysis.

## ACS Metamorphosis in COVID-19 Era

Now that the control of COVID-19 contagion and management is improved, with resulting lower rates of morbidity, it is time for clinicians to look beyond COVID-19 and to care about the cardiovascular consequences of the pandemic. A serious concern regarding ACS is currently affecting global healthcare services: a downward trend in ACS incidence has been registered all over the world, awakening the interest of the scientific community. First, the Italian society of Cardiology multicenter register, which compared acute MI incidence in a week with the equivalent period in 2019, observed a drastic reduction of 48.4% (*p* < 0.001), which was significant for both STEMI (26.5%, higher for women: 41.2% vs. 17.8%) and NSTEMI (65.1%) and was similar throughout Italy (52.1% Northern vs. 59.3% Central vs. 52.1% Southern). Importantly, they have also registered a substantial increase in STEMI fatality rate [risk ratio (RR) = 3.3, 1.7–6.6; *p* < 0.001] and complications (RR = 1.8; 1.1–2.8; *p* = 0.009) during the pandemic, compared to 2019 (46).

Then, Metzler et al. conducted an Austrian nationwide retrospective survey involving 17 primary PCI centers for 27 days during COVID-19 outbreak, founding a relative reduction from the beginning to the end of this period of 39.4% in admission for all subtypes of ACS (47). Huet et al. reported almost halved numbers of admission for acute MI or heart failure in 9 French ICU centers comparing 14 days periods before and after containment (4.8±1.6 vs. 2.6±1.5 patients per day, *p* = 0.0006) (48).

Furthermore, the impact of the pandemic on interventional cardiology procedures has been assessed by Garcia et al., who quantified STEMI activations in 9 high-volume (>100 PCI/year) United States cardiac Cath-labs from January 1, 2019, to March 31, 2020, and observed a 38% reduction in Cath-lab STEMI activations in the after-COVID period (49), similar to the 40% registered in Spain (50). Moreover, an analysis of the Italian Society of Interventional Cardiology (GISE) reported a decrease in interventional coronary and structural procedures of 48.5% for ICA, 45.7% for PCI, 84.7% for transcatheter aortic valve replacement, and 50% for Mitraclip in Piedmont (Italy), during the COVID-19 period (51).

In our experience, we have observed not only a reduction of hospitalization for AMI but also a dramatic increase of hospitalization for subacute myocardial infarction >72 h, with cases of malignant arrhythmias and severe heart failure resistant to conventional therapy and often requiring inotropic support; this unavoidably resulted in poor prognosis for patients and challenges for clinicians to select the best therapeutic strategy, due to the doubtful benefits of a late revascularization and the difficult selection of patients for the allocation of advanced therapeutic resources (such as mechanical assist devices).

### Causative Factors

Altogether, these data depict a picture of almost half of patients with ACS not reaching the hospital and not receiving timely treatment. The embraceable opinion is that this worrisome phenomenon could be multifactorial:

❖ **Patient-related factors:** to start with, there was a reduced referral to ED of patients with chest discomfort or unclear ACS symptoms due to their fear of catching SARS-CoV-2 in the hospital, encouraged by in-hospital contagion described by the media and by the strict instructions to stay at home. These have led patients to underestimate their symptoms, such as in a case-report by Masroor et al. regarding a 48-year-old man who referred to the ED for chest pain started 2 days earlier, but not seeking attention until later, due to his reluctance to access the hospital for dreaded COVID-19 contagion. ECG clearly showed STEMI and he underwent ICA with successful PCI on the occluded right coronary artery; few hours later, he developed cardiogenic shock for postinfarction ventricular septal defect of 2 cm, initially treated with intra-aortic balloon pump to let the myocardium heal, and then with surgical repair using a pericardial patch (52). Other patient-related features explaining the reduction in hospital admissions for ACS during the COVID-19 era are a negative psychological response, emotional distress, distrust/avoidance behaviors, and reluctance to activate pre–hospital networks.
❖ **Healthcare-related factors**: during this period, the emergency services have focused on COVID-19, with most healthcare resources relocated to manage the pandemic and with possible fails in identification of MI, which could have led to an artificial decreasing of ACS diagnoses. First, the priority given to COVID-19 suspected or known patients could have finally distracted from cardiovascular emergencies. Then, it seems that, for patients presenting symptoms consistent with COVID-19, all the resources and clinical attention have been dedicated to excluding SARS-CoV-2 infection, with consequent overlooking of acute cardiovascular conditions, causing misdiagnosis and/or delayed treatment. A clear example was described by Yousefzai et al. in a case-report of a 56-year-old patient with cardiovascular risk factors presenting exertional dyspnea and left bundle branch block at ECG who at first hesitated to refer to the ED and was then misdiagnosed with COVID-19-induced acute myocarditis, though presenting STEMI. Meanwhile, he developed acute respiratory distress syndrome requiring ventricular assistance and underwent late ICA with evidence of 99% left anterior descending coronary stenosis, 60% proximal circumflex artery stenosis, and moderate disease on right coronary artery; therefore, the clinicians opted not to perform revascularization. Then, after this completed anterior MI, he remained in ICU waiting for recovery or definitive ventricular assistance therapy (53).

### Short- and Long-Term Consequences

The delay among symptoms presentation and revascularization could result in dramatic effects. Noteworthy, conjunction of the longer time from symptoms onset to first medical contact due to patients' reticence and waiting times for triaging, COVID-19 testing (since not all the healthcare facilities are equipped with ultra-rapid tests) and personnel precautions, would result in further delay for a needed PCI. This should represent an alarm for clinicians and public health, since the paramount importance of the timing of primary revascularization to save myocardial structure and function is well-known (53). In fact, in a recent study by Trabattoni et al., despite a regional optimization of the STEMI network through a re-structured Hub-Spoke model in Lombardy (Italy), a significant delay (> 24 h) in patients' referral to ED was present in 41% of STEMI patients in 2020, compared to 20% in 2019, resulting in in-hospital mortality rates of 38 vs. 10%, respectively (54). Similar results were shown by a Chinese group in an observational study on 149 patients with MI before *(group 1, n* = *85 patients)* and after *(group 2, n* = *64 patients)* COVID-19 emergency measures; the second group not only had longer symptom-to-first medical contact time and higher presentation rates out of the PCI window (33 vs. 27.8%) but also showed a more elevated incidence of the composite outcome measure including in-hospital death, cardiogenic shock, sustained ventricular tachycardia/fibrillation, and use of mechanical circulatory support (29.7, vs. 14.1%, *p* = 0.02) (55).

**Figure 2:**
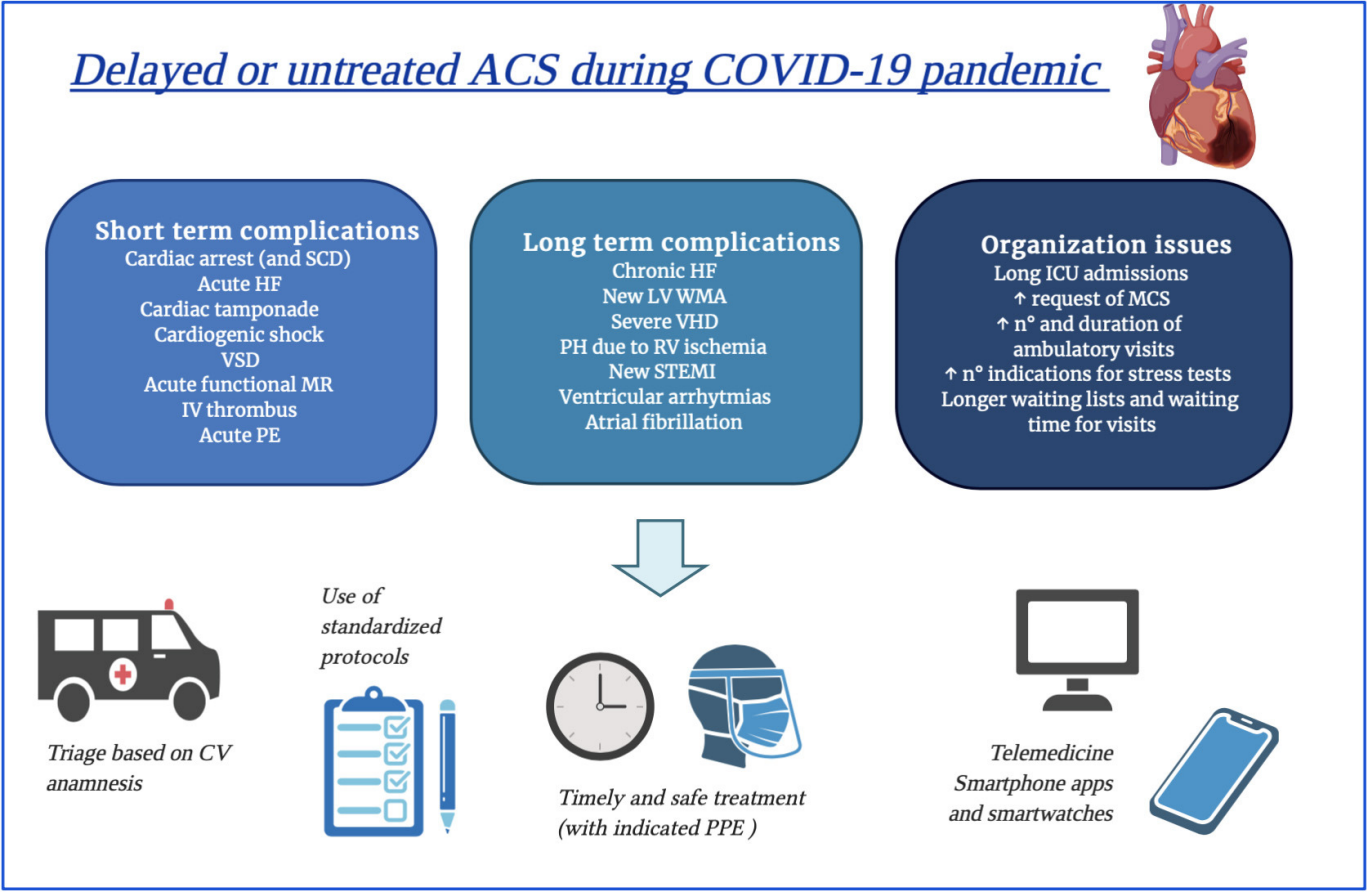
Possible complications deriving from late or untreated acute coronary syndromes during COVID-19 pandemic and figurative hints for limiting them. ACS; acute coronary syndromes; HF, heart failure; ICU, intensive care unit; IV, intraventricular; LV, left ventricular; MCS, mechanical circulatory support; NSTEMI, non-ST elevation myocardial infarction; PE, pulmonary embolism; PH, pulmonary hypertension; PPE, personal protective equipment; RV, right ventricular; SCD, sudden cardiac death; STEMI, ST elevation myocardial infarction; VHD, valvular heart disease; VSD, ventricular septum defect; WMA, wall motion abnormalities.

These data, together with those previously mentioned (34), suggest that an increase in the incidence of late presenting MIs with chronic heart failure and sudden cardiac death is the most expectable eventuality in the near future, together with raised early and late morbidity and mortality. Short term-complications would require prolonged hospitalization in ICU, which could represent a serious concern in these times of poor resources. Over the long-term, suboptimal revascularization and large infarct size will result in maladaptive ventricular remodeling and dysfunction (56). Short and long-term complications and their impact on healthcare services are presented in Figure 2.

The earliest reports referred to cases with initially mild symptoms who experienced sudden cardiac death at home while in quarantine (57). Moreover, Baldi et al. described an increased incidence of out-of-hospital cardiac arrest during 40 days of COVID-19 pandemic in Italy compared to the same period in 2019, which such cumulative increased incidence being strongly associated with the diffusion of COVID-19 (58). Similarly, a 4.97-fold increase in out-of-hospital sudden cardiac arrest and a doubling of pronounced deaths on the scene was reported in New York City during the surge of pandemic, compared with the same period (March 20–April 22) of 2019 (59). These data could reflect the eventual consequences of medical care avoidance or distraction.

### Possible Solutions

As the ESC guidance for the diagnosis and management of cardiovascular disease during the COVID-19 pandemic illustrates (60), it would be rational to triage patients with suspected or known COVID-19 according to the presence of underlying cardiovascular risk factors and co-morbidities, as well as to evidence of myocardial injury, in order to select those who deserve prioritized treatment and even more aggressive therapeutic strategies.

Organization of healthcare facilities should be improved with dedicated pathways and rapid SARS-CoV-2 testing, if available, allowing a timely supply of diagnostic and interventional procedures. ACS patients with highly suspected COVID-19 should be isolated and undergo necessary laboratory and imaging tests, with all healthcare workers wearing the appropriate PPE (34).

Besides, the most important issue is to educate the general population about the early recognition of high-risk ACS symptoms with promptly referral to ED (or at least to contact a physician) in such cases. This could be reached by social media, television, and journals. Interestingly, following this rationale, the Italian Society of Cardiology promoted a national campaign to raise public awareness about MI symptoms during the outbreak, showing encouraging results in terms of subsequent fall in the time from symptoms to ED admission (50).

Social education should emphasize the concept of an outweigh of untreated-MI consequences, rather than of COVID-19 in-hospital infection, since hospitals are now equipped with appropriate PPE and follow the preventive protocols to minimize the risk of contagion. The use of telemedicine and/or telemonitoring in doubtful cases would allow to obtain a close follow-up of patients' symptoms and clinical conditions and, sometimes, to perform some kinds of triaging in order to avoid unrecognized MI on one hand, and to optimize resources allocation on the other hand. More compliant patients could also be engaged in the use of smartwatches and smartphone apps, achieving rapid medical screening and/or self-monitoring.

## Conclusions

During the COVID-19 pandemic, the topic of ACS has been widely discussed. Even if there is paucity of randomized data on the best methods for management, expert consensus and international society recommendation could help us in adopting a standardized approach. First of all, it is important to distinguish between primary ACS or COVID-AMI and, for the latter, discriminate the actual etiology and provide the optimal treatment. This should be done balancing timeliness of screening and conscious use of diagnostic resources and protective measures, in order to ensure safety conditions to all patients and healthcare professionals. COVID status should be assessed as soon as possible. Each primary PCI center should evaluate the feasibility of a timely primary PCI, based on staff, PPE and Cath-lab availability, and the need for additional testing. Otherwise, a first approach with fibrinolysis should be considered. The other important concern is the global registration of lower rates of admitted (and therefore treated) patients with ACS. This could lead to a substantial increase in early and late infarct-related morbidity and mortality. To face the possible collateral cardiac damage caused by COVID-19, every attempt should be done by the clinicians in means of avoiding delayed or missed diagnosis, re-organization of healthcare tools, and social education.

## Author Contributions

MC, MCP, GM, FD'A, PC, GP, GB, and MF performed the data search and drafted the manuscript. MC, FF, GP, SM, and SV critically revised the draft. All Authors contributed to the conception of this work and approved the final version of the manuscript.

## Conflict of Interest

The authors declare that the research was conducted in the absence of any commercial or financial relationships that could be construed as a potential conflict of interest.
